# Transient pure red blood cell aplasia as clinical presentation of congenital hemolytic anemia: a case report

**DOI:** 10.4076/1757-1626-2-6814

**Published:** 2009-06-17

**Authors:** Sofia Figueiredo, Daniela Pio, Margarida Martins, Carlos Seabra, Marisol Pinhal, Arménia Parada

**Affiliations:** 1 Pediatric Department, Hospital Infante D. PedroEPE, Avenida Artur Ravara, 3814-501 AveiroPortugal; 2 Hematology Unit, Hospital Infante D. PedroEPE, Avenida Artur Ravara, 3814-501 AveiroPortugal

## Abstract

Hereditary elliptocytosis is a congenital hemolytic anemia characterized by the presence of oval shaped erythrocytes in the peripheral blood. In rare cases, transient pure red blood cell aplasia can be the initial clinical presentation.

We report a case of a 27-month-old boy admitted with fever without focus, severe poikilocytic anemia, no evidence of hemolysis, a normocelular bone marrow and negative serological tests for viral infections. One month before admission, he had been treated with phenytoin and valproate after a seizure episode without fever.

Analysis of red cell membrane proteins showed a 16% decrease in spectrin levels, also detected in his father and brother, confirming the diagnosis of elliptocytosis.

Only his father carried the α^LELY^ mutation, in trans to the α-spectrin mutation.

## Introduction

Hereditary elliptocytosis (HE) is a congenital hemolytic disorder characterized by an elongated (cigar or oval) shape of erythrocytes [1-3].

This disorder is the result of a defect in one of the cytoskeletal proteins in the red blood cells membrane that are usually responsible for the elasticity and durability of circulating erythrocytes. A few mutations of the alpha-spectrin subunit are responsible for most cases of HE. HE also occurs with deficiencies in protein 4.1 or glycophorin C or when defects of band 3 protein or beta-spectrin subunit impair ankyrin binding [1]. Although spontaneous mutations have been reported, HE is transmitted predominantly as an autosomal dominant trait with at least 4 genetic loci implicated [1,2,4]. It is characterized by clinical, biochemical, and genetic heterogeneity.

The true incidence is unknown because most of the patients are asymptomatic but generally it is estimated to be present in 1 per 2000-5000 individuals [1-3]. HE has no sex predilection [1,2].

The clinical presentation of patients with HE is highly variable. Most patients are asymptomatic, and the diagnosis is made incidentally when a blood smear is examined. Less often, patients experience intermittent episodes of more intense hemolysis with anemia, jaundice, and splenomegaly [1].

Typical presenting signs in patients with severe HE include neonatal hyperbilirubinemia and anemia in the first few months of life. In addition, they present frequently complications of severe hemolysis, including anemia, splenomegaly, growth retardation, frontal bossing, and early gallbladder disease [1].

Members of the same family may exhibit different clinical courses, and an individual's frequency and severity of hemolysis may change with time [1,5].

Transient pure red blood cell aplasia has been reported in patients with chronic hemolysis such as HE. Under these conditions, the erythrocytes’s life span is much shorter than in normal individuals so that a brief cessation of erythropoiesis may cause severe anemia, known as “aplasic crisis” [5,6]. Patients can develop uncompensated anemia with marked weakness and dyspnea, pallor, tachycardia and incipient heart failure. The etiology of pure red cell aplasia is diverse, with parvovirus being the most common cause [1,5]. Other causes identified: 1) viral infections like mumps, infectious mononucleosis, atypical mycoplasmal pneumonia and viral hepatitis; 2) drugs: antiepileptic medications (eg. phenytoin, carbamazepine, sodium valproate), azathioprine, chloramphenicol, sulfonamides, isoniazid, procainamide [5]. In some cases a bone marrow aspiration may be performed, which typically shows erythroid hypoplasia or aplasia and characteristic large proerythroblasts confirming the diagnosis. However if it is done during the recovery phase it may show active erythropoiesis that can be misleading.

The hallmark of HE is the presence of cigar-shaped elliptocytes on the peripheral blood smear [1,2,7]. Elliptocytes are normochromic and normocytic and range from few to 100% of the erythrocytes [1]. Spherocytes, ovalocytes, stomatocytes, microcytes and fragmented cells may also be observed [1,7]. The elliptical erythrocyte form is acquired in the circulation and that is why the reticulocytes and bone marrow red blood precursors are normal in shape [2].

A complete blood count (CBC) reveals the degree of anemia. The reticulocyte count reflects the severity of hemolysis - in mild HE is typically less than 5%, but in the severe forms of HE reticulocyte counts can be as high as 30% [1].

A review of the family history and the analysis of the red blood cell morphology can usually confirm the diagnosis [1,7]. Sometimes additional tests may be required including the analysis of cDNA and genomic DNA and the study of membrane proteins by gel electrophoresis and spectrin-dimer self-association assays [1].

Treatment is rarely indicated for patients with mild HE. In severe cases, occasional erythrocyte transfusions may be required. Daily folate is recommended for patients with significant chronic hemolysis [1,7]. Phototherapy and exchange transfusion are warranted in cases of severe anemia and hyperbilirubinemia. Splenectomy has been palliative in severe cases of HE, but should be avoided in patients younger than 5 years old due to the risk of pneumococcal disease.

Here we report a case of transient pure red blood cell aplasia as clinical presentation of HE in a patient with no history or laboratory evidence of hemolysis, who had been treated with phenytoin and valproate after a seizure episode without fever one month before admission.

## Case presentation

A 27-month-old Caucasian boy was admitted to the pediatric unit with fever (39.5°C) for the last 3 days, poor peripheral perfusion, myalgias and anorexia. Twelve days before he had had a cold-like syndrome. Physical examination revealed intense pallor, a runny nose and bilateral cervical lymph node enlargement. He had no jaundice.

He had been admitted one month before with seizures without fever and a diagnosis of benign transient hyperphosphatasemia of childhood was then established. He was treated with phenytoin replaced shortly thereafter by sodium valproate, which he took for 3 days. During this admission he had no anemia nor any clinical or laboratory evidence of hemolysis.

He is the second son of a non-consanguineous couple with unremarkable family history; there were no records of anemia, jaundice, gallstones or splenomegaly. He required phototherapy for neonatal jaundice.

Laboratory studies (Table 1) showed severe microcytic and hypochromic anemia with reticulocytopenia, a normal white blood cell count and thrombocytosis. Peripheral blood smear (PBS) showed anisocytosis and some cigar-shaped cells. Serum CRP levels were high (29.1 mg/dl, normal < 0.5 mg/dl). Additional laboratory studies, including serum bilirubin, LDH, and a Coombs test, were normal with no evidence of hemolysis.

**Table 1.** Evolution of laboratory data

	D1	D5	D12	D29
Hb (g/L)	51	48	86	101
MCV (mm^3^)	67	68	78	74
MCH (pg)	22	22	26.7	23.6
RDW (%)	15.8	15.1	34.4	31.7
Leukocytes (*10^9^/mm^3^)	11100	5400	18.9	24.9
Neutrophilis	4040	1140	6900	5500
Lynfocytes	5640	3450	2280	1570
Platelet (*10^3^/mm^3^)	530	597	578	617
CRP (mg/dl)	29.1	3		
Reticulocytes (%)	0.9	3.69	10.6	1.33
Bilirubin				
Total bilirubin (mg/dl)	0.32	0.26	0.41	0.30
Direct bilirubin (mg/dl)	0.06	0.04	0.12	0.05
LDH		266	292	278
direct and indirect coombs	Negative			

Legend: Hb - Hemoglobin, MCV - mean corpuscular volume, MCH - mean corpuscular hemoglobin concentration, RDW - Red Cell Distribution Width, CRP - C-reactive protein, LDH - Lactate dehydrogenase.

A bone marrow aspirate revealed a normocelular marrow with mild dyseritropoiesis, but with normal hematopoiesis, having no blasts or other abnormal cells, and increased iron in the reticuloendothelial system with no ringed sideroblasts.

Serological tests for mycoplasma, hepatitis, rubella, cytomegalovirus, toxoplasmosis, Epstein-Barr virus, parvovirus (B19) and adenovirus infection were negative. Blood and urine cultures were also negative.

Chest radiography and abdominal sonography were normal.

After 3 days of intravenous ceftriaxone he became apyretic. A rise of Hb and reticulocyte levels was observed by the fifth day of admission and there was no need for red blood cell transfusions. A follow up PBS showed microcytosis, anisopoikilocytosis, polychromasia, teardrop cells, fragmented cells and elliptocytes. Three weeks later, the serological tests for viral infections were again negative.

Hemoglobin electrophoresis, enzymes studies, high performance liquid chromatography, electrophoresis of red cell membrane proteins and α^LELY^ mutation studies were carried out in the patient and his family. These studies showed a 16%, 15% and 18% decrease in spectrin levels in the patient, his brother and his father, respectively, confirming the diagnosis of hereditary elliptocytosis. Only the father is heterozygous for the α^LELY^ mutation trans to α-spectrin mutation.

## Discussion and conclusion

The patient presented with severe anemia, no clinical or laboratory evidence of a hemolytic process, and no family history of hemolysis. Reticulocyte count was low for the degree of anemia and compatible with medullar compromise. PBS initially showed severe poikilocytosis that could be explained by some degree of stress erythropoiesis. A normocellular bone marrow aspirate with normal hematopoiesis, no blast, no abnormal cells, or ringed sideroblast excluded acute leukemia or other neoplastic diseases involving the marrow, aplastic anemia, or sideroblastic anemia, but could be interpreted as the recovery phase of red cell aplasia.

Normal hemoglobin values one month before admission suggested a somewhat rapid process, and did not agree with a diagnosis of transient erythroblastopenia of childhood [8].

The history of neonatal jaundice and the previous cold-like syndrome, coupled with the PBS findings of severe poikilocytosis were also compatible with transient pure red blood cell aplasia in recovery in a patient with some form of congenital hemolytic anemia.

Pyropoikilocytosis was included in the differential diagnosis and a search for α^LELY^ mutations was added to other red cell membrane protein studies. Later smears showed more typical elliptocytes. Most patients with elliptocytosis are asymptomatic. Therefore, the unremarkable family history of our patient is not unexpected. The electrophoresis of the membrane proteins showed that the patient, his father and brother have a decrease in spectrin levels compatible with hereditary elliptocytosis. Additionally, his father carried the α^LELY^ mutation, in trans to the α-spectrin mutation.

Several possibilities exist in this case that could explain the aplastic crisis. An infectious cause agrees with a history of a cold-like syndrome a few days before admission, an initial presentation with fever and a positive serum CRP, but blood cultures and extensive serological tests were negative.

Other possible causes include the drugs phenytoin and sodium valproate, taken one month before. This etiology has been reported, but the drugs were used only for a short time.

No published report in the literature exists to our knowledge that correlates pure red cell aplasia with benign hyperphosphatasemia of childhood. One-half of aplastic crises cases remain unexplained [9].

In these cases of red cell aplasia, a transfusion therapy may be necessary if symptomatic anemia, the offending drugs should be discontinued, and the associated infections or other illness should be treated. Our patient presented an intense pallor without signs of clinical decompensation (like hemodynamic instability) in spite of low Hb level. In addition, bone marrow aspirate evidence signs of marrow recovery. Hence, we decided to make a close follow-up with blood cell count instead of transfusion therapy. The patient was also treated for a possible bacterial infection in spite of negative blood culture. In admission, he was not taking any drugs that could be responsible for aplasia.

The publication of this case has the objective to alert for the possibility of chronicle hemolytic anemia to be presented as an acute self-limited pure red cell aplasia, which may be due to drugs.

## List of abbreviations

CBC: Complete blood count
CRP: C-Reactive protein
DNA: Deoxyribonucleic acid
HE: Hereditary elliptocytosis
LDH: Lactate dehydrogenase
PBS: Peripheral blood smear
